# Polycomb Domain Formation Depends on Short and Long Distance Regulatory Cues

**DOI:** 10.1371/journal.pone.0056531

**Published:** 2013-02-20

**Authors:** Bernd Schuettengruber, Giacomo Cavalli

**Affiliations:** Institut de Génétique Humaine, Centre National de la Recherche Scientifique, Montpellier, France; University of Nevada School of Medicine, United States of America

## Abstract

**Background:**

Polycomb group (PcG) proteins dynamically define cellular identities through the epigenetic repression of key developmental genes. In *Drosophila, cis*-regulatory regions termed PcG response elements (PREs) act as nucleation sites for PcG proteins to create large repressive PcG domains that are marked by trimethylation of lysine 27 on histone H3 (H3K27me3). In addition to an action in *cis,* PREs can interact over long distances, thereby enhancing PcG dependent silencing. How PcG domains are established, which factors limit their propagation *in cis*, and how long range interactions of PREs *in trans* affect the chromatin structure is largely unknown.

**Principal Findings:**

We demonstrate that the insertion of a PRE-containing transgene in the *Drosophila* genome generates an artificial PcG domain and we analyze its organization by quantitative ChIP and ChIP-on-chip experiments. Intriguingly, a boundary element and known insulator proteins do not necessarily interfere with spreading of H3K27me3. Instead, domain borders correlate with the presence of promoter regions bound by RNA Polymerase II and active chromatin marks. In contrast, genes that are silent during early fly development get included within the PcG domain and this incorporation interferes with gene activation at later developmental stages. Moreover, *trans-*interaction of the transgenic PRE with its homologous endogenous PRE results in increased PcG binding, correlating with reinforced silencing of genes within the domain borders.

**Conclusions:**

Our results suggest that higher-order organization of PcG-bound chromatin can stabilize gene silencing within PcG domains. Further we propose that multi-protein complexes associated with active promoters are able to define the limits of PcG domains. Future work aimed to pinpoint the factors providing this barrier function will be required to understand the precise molecular mechanism by which active promoter regions can act as boundaries to stop spreading of H3K27me3.

## Introduction

Polycomb-group proteins (PcG) proteins were initially discovered in flies for their role in maintaining the repressed state of homeotic (HOX) genes in appropriate spatial domains, after the segmentation gene products have disappeared in early embryonic development. In addition to their function as cellular memory system, PcG factors dynamically regulate cell fate decisions and control cell proliferation [1], [2].

Biochemical studies showed that PcG proteins exist in distinct large multiprotein complexes [3], [4]. One of them is the PRC2 complex, which contains histone methyltransferase activity specific for lysine 27 on histone H3 (H3K27me3), catalyzed by the SET-domain-containing protein Enhancer of Zeste, E(z) [5]. This hallmark of PcG-dependent gene silencing is specifically recognized by the chromodomain of the Polycomb protein (PC) [6], a component of the PRC1 complex which mediates ubiquitylation of K119 of histone H2A by the dRING E3 ligase [7]. The role of PcG-associated histone modifications in gene silencing is still not fully understood. PRC2-mediated H3K27me3 may directly interfere with transcriptional activation or may inhibit the deposition of activating histone marks [7]. On the other hand, H3K27me3 may primarily serve to recruit PRC1 complexes to their target chromatin, building up a chromatin structure that interferes with ATP-dependent nucleosome remodelling activities or RNA Polymerase II (RNA Pol II) recruitment [6]. However, since it has been shown that RNA Pol II can be recruited to at least a subset of PcG target genes [8] and H2AK119 ubiquitylation has been suggested to interfere with transcriptional elongation [9], PRC1 may also silence by acting downstream of RNA Pol II assembly at promoters.

In *Drosophila*, PcG complexes are recruited to chromatin via regulatory DNA elements called PcG response elements (PREs) [2]. These sequences can drive epigenetic inheritance of silent chromatin states throughout development and are sufficient in a transgenic context to silence reporter genes in a PcG-dependent manner [10]. In the endogenous context they act as nucleation sites for PcG proteins to form large repressive domains marked by H3K27me3 that are often hundreds of kilobases (kb) in size, the largest ones including the Hox gene clusters [11]–[18]. These large PcG domains could provide the basis of a robust epigenetic memory to maintain gene expression states through mitosis or DNA replication.

Functionally it has been proposed that spreading of the repressive histone mark H3K27me3 is blocked by insulator proteins. Binding sites for the insulator protein Su(Hw) have been shown to interfere with the spreading of the H3K27me3 mark [19], [20]. In addition, genome wide mapping studies of known insulator proteins, including CTCF (CCCTC-binding factor), GAGA factor (GAF), Mod(mdg4), BEAF-32 and CP190 revealed enrichment of all proteins at H3K27me3 borders [21]–[23]. However the importance of insulator elements in the establishment and maintenance of H3K27me3 borders is under intense discussion: It has been previously reported that knock down of CP190/CTCF causes spreading of H3K27me3, supporting their function as chromatin boundary elements [24], [25]. However, a recent study demonstrated that knockdown of insulator proteins results in a reduction of H3K27me3 levels within repressed domains without H3K27me3 spreading, suggesting that insulators might play a role in the maintenance of silent chromatin in PcG domains [23]. Another genomic feature that correlates with the presence of H3K27me3 borders are promoter regions associated with active chromatin marks and RNA Pol II [18], [22], [24]. Therefore transcriptional activity itself might be sufficient to prevent spreading of the H3K27me3 mark. This model is supported by a recent report demonstrating that insulators restrict the spreading of H3K27me3 only at a small number of H3K27me3 domains, whereas H3K27me3 borders associated with promoter regions of active genes are not affected after loss of function of insulator proteins [22]. This suggests that insulator elements only contribute to the establishment of a subset of H3K27me3 borders, whereas they are dispensable for their maintenance in most cases.

Importantly, the action of PREs to form large repressive H3K27me3 domains is not restricted to the linear chromatin fiber: PREs can interact at long distances [26], thereby forming physical domains enriched for repressive histone marks. Interestingly, recent interrogation of chromosome architecture in Drosophila embryos revealed that insulator proteins and active histone marks are enriched at the borders of these physical domains, emphasizing their importance in demarcating physical boundaries of PcG repressed domains [27]. Cytological analysis of the location of PRE-target genes demonstrated that they cluster in the nucleus at sites of high concentrations of PcG proteins (PcG bodies), which also correspond to physical sites of gene silencing [28], [29]. This suggests that interactions between PRE-containing regions may be relevant for gene regulation [30], providing an additional regulatory layer to PcG-mediated gene silencing. However, the effect of PRE clustering on PcG-mediated histone modifications and PcG domain topology has not been studied.

Using a previously established transgenic fly line [31], we generated a PcG domain at a chromosomal region that is normally devoid of these proteins and asked which chromatin features interfere with the linear spreading of H3K27me3 and how long range interaction with its homologous PRE impacts PcG-associated chromatin marks and gene silencing. We found that an insulator element and recently published binding sites for known insulator proteins [21] do not necessarily interfere with spreading of H3K27me3, whereas promoters marked by RNA Polymerase II and active chromatin components delimit the repressive H3K27me3 domain, supporting the idea that active promoters can act as PcG domain barriers. In contrast, promoter regions of genes that are silent during early fly development get incorporated within the PcG domain and this interferes with their activation at later developmental stages. Further, we demonstrate that long distance interactions of homologous PREs do not affect the chromatin boundaries of the PcG domain, but enhance PcG binding and repression of genes within the domain, suggesting that multiple PREs may interact to achieve a stronger stability of Polycomb-mediated silencing.

## Results

### A Transgenic PRE Induces Spreading of H3K27me3 into Flanking Genomic Regions, Creating an Artificial PcG Domain

**Figure 1:**
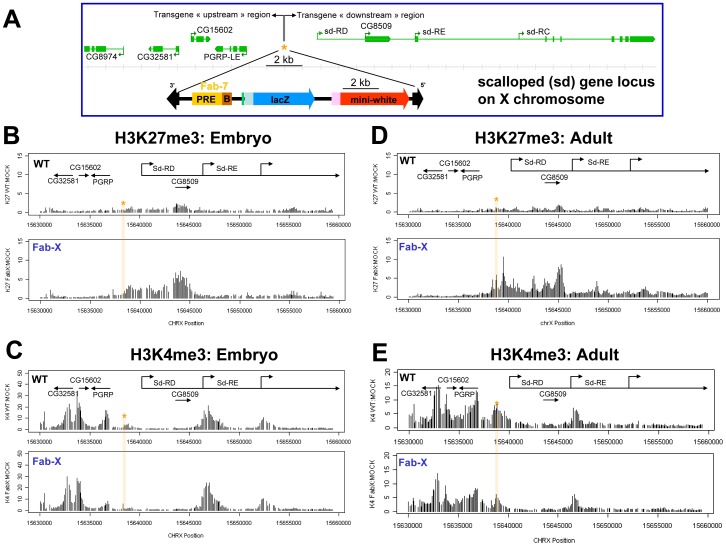
Spreading of H3K27me3 into flanking genomic regions after insertion of the *Fab-7* transgene at the *sd* gene locus. (A) Schematic representation of the *Fab-7* containing transgene and the *scalloped* (*sd*) gene locus on the X chromosome. Orange asterisk represents the transgene insertion site. Note that the boundary region of the *Fab-7* element is located between the PRE and the reporter genes. (B) and (C) ChIP-on-chip analysis of the *sd* gene locus from 4–12 hours old embryos of indicated fly lines. Fold changes between IP with H3K27me3 antibodies (B) or H3K4me3 antibodies (C) and mock IP are plotted on the Y axis. Annotated genes are shown on the top of the panel. Orange asterisk indicates the transgene insertion site. (D) and (E) ChIP-on-chip analysis of *sd* gene locus from female adult flies in the indicated fly lines. Fold changes between IP with H3K27me3 antibodies (D) or H3K4me3 antibodies (E) and mock IP are plotted on the Y axis. Annotated genes are shown on the top of the panel. Orange asterisk indicates the transgene insertion site. Note that H3K27me3 can spread into downstream regions of the transgene insertion site until the *sd*-RE promoter region, which is marked by H3K4me3. No significant levels of H3K27me3 can be observed upstream the transgene insertion site. Levels of H3K4me3 do not change significantly after insertion of the *Fab-7* containing transgene.

*Fab-7* is a well-defined chromosomal element that contains a PRE and is involved in the regulation of the homeotic gene *Abdominal-B* (*Abd-B*) within the homeotic bithorax complex (BX-C) [32]. A transgene containing a 3.6 kb fragment from the *Fab-7* region, upstream of a *mini-white* reporter and inserted at the *scalloped* (*sd*) locus of the X chromosome (referred to as the Fab-X line) has been previously shown to ectopically recruit PcG proteins [31] (Figure S1), inducing silencing of both the *mini-white* eye color reporter gene and of the endogenous *sd* gene downstream of *Fab-7* [28]. However the effect of the transgenic PRE on the surrounding chromatin has not been investigated so far. The *Fab-7* containing transgene is inserted 1.6 kb upstream of the *scalloped*-RD (*sd*-RD) promoter (Figure 1A). We analyzed embryonic chromatin surrounding the transgene insertion site from the Fab-X line by ChIP-chip. We found that insertion of the *Fab-7* containing transgene at the *sd* gene locus results in spreading of H3K27me3 downstream of the transgenic PRE into flanking genomic regions that are not significantly methylated in wild type (WT) embryos (Figure 1B). H3K27me3 propagates downstream up to the promoter of the *sd*-RE transcript (about 7 kb away from the transgene insertion site and at 18 kb total distance from the position of the *Fab-7* PRE), where its levels significantly decrease close to background levels. Intriguingly, spreading is unidirectional, since no increased H3K27me3 levels were found upstream of the transgene insertion site (Figure 1B). ChIP-chip assays on adult flies revealed a similar asymmetric spreading of H3K27me3 (Figure 1D). Although the size and domain borders of the H3K27me3 domain is identical in adult flies and embryos, more pronounced peaks of H3K27me3 were found in adult flies. Intriguingly, these peaks correlate well with promoter regions of genes downstream of the transgenic PRE.

### Confinement of PcG Domains by Promoters Marked by Active Chromatin Marks

Next we asked why spreading is unidirectional and what prevents the coating of a larger region by the H3K27me3 mark. We considered two parameters: chromatin boundaries or insulators, and the presence of active chromatin components. The proximal end of the 3.6 kb *Fab-7* fragment contains a so-called boundary element, which has been shown to be essential to keep the iab-6 and iab-7 *cis*-regulatory domains of the BX-C autonomous [32]. This boundary sequence could, in principle, stop the spreading of H3K27me3 in one direction. However, in the case of the Fab-X line the boundary is located between the *Fab-7* PRE and the *sd* gene downstream of the transgene insertion site (Figure 1A). Since we observed spreading of H3K27me3 in this direction, this suggests that the *Fab-7* boundary element does not interfere with the propagation of repressive histone marks. This observation could be confirmed in another transgenic fly line where the *Fab-7* element is cloned in the reverse orientation upstream of the *mini-white* marker gene and is inserted at a different chromosomal location (data not shown).

To examine the effect of endogenous insulator proteins in blocking spreading of the H3K27me3 mark at the transgenic gene locus, we compared the genomic location of the domain borders of the ectopic PcG domain with the previously published distribution of six insulator proteins at the *sd* locus in wild type *Dosophila* embryos [21] (Figure S2). In contrast to many endogenous domains, no double occupancy of CTCF and CP190 is found to be associated with genomic sites marking the ectopic domain borders [24]. Moreover, no significant binding of SuHw can be detected at the sd gene locus close to the transgene insertion site, whereas peaks of GAF and BEAF32 can be detected at promoter regions (PGRP-LE and sd-RE) demarcating the ectopic domain (Figure S2). These proteins could in theory act as chromatin boundaries. However, another BEAF32 binding site colocalized with CP190 at a genomic side that becomes covered by H3K27me3 (upstream of the CG8509 promoter region) does not interfere with H3K27me3 spreading. This indicates that, if BEAF32 does act as barrier for H3K27me3 spreading further downstream at the sd-RE promoter, additional factors are required for its boundary activity. To test a possible role of GAF in the boundary function we crossed the Fab-X line with the Trl^R85^ allele, a null mutant for GAF [33], and analyzed the progeny heterozygous for the GAF mutation for increased silencing of the scalloped gene. However, we did not observe a stronger scalloped mutant phenotype that would indicate increased silencing of the sd gene, as one would expect in the case of increased spreading of H3K27me3 over the sd gene locus (data not shown).

We next compared the extent of spreading of the artificial PcG domain with the distribution of the H3K4me3 mark at the *sd* gene locus. Immediately upstream of the transgene insertion site is the promoter of the gene *PGRP*-LE that is highly tri-methylated at H3K4, both at the embryonic stage (Figure 1C) and in adult flies (Figure 1E). In this direction, no major chromatin changes could be detected. Downstream of the *Fab-7* transgene, two promoters of the *sd*-RD transcript and the *CG8509* gene are not tri-methylated on H3K4 and do not interfere with spreading of repressive marks. In contrast, the next promoter (of the *sd*-RE transcript) is tri-methylated on H3K4 and is adjacent to the *Fab-7*-mediated H3K27me3 domain boundary (Figure 1B and 1D). Importantly, we noticed that the boundaries of the H3K27me3 domain do not precisely overlap with H3K4me3 peaks at the transcriptional start site (TSS) of the bordering genes. Instead H3K27me3 levels already drop at the promoter region of the corresponding genes a couple of hundred base pairs upstream of the H3K4me3 peak, suggesting that promoter-associated protein complexes, rather than the activating histone mark, may interfere with the spreading of the repressive histone mark.

### Transcriptional Activity is not Essential to Block Spreading of H3K27me3

**Figure 2:**
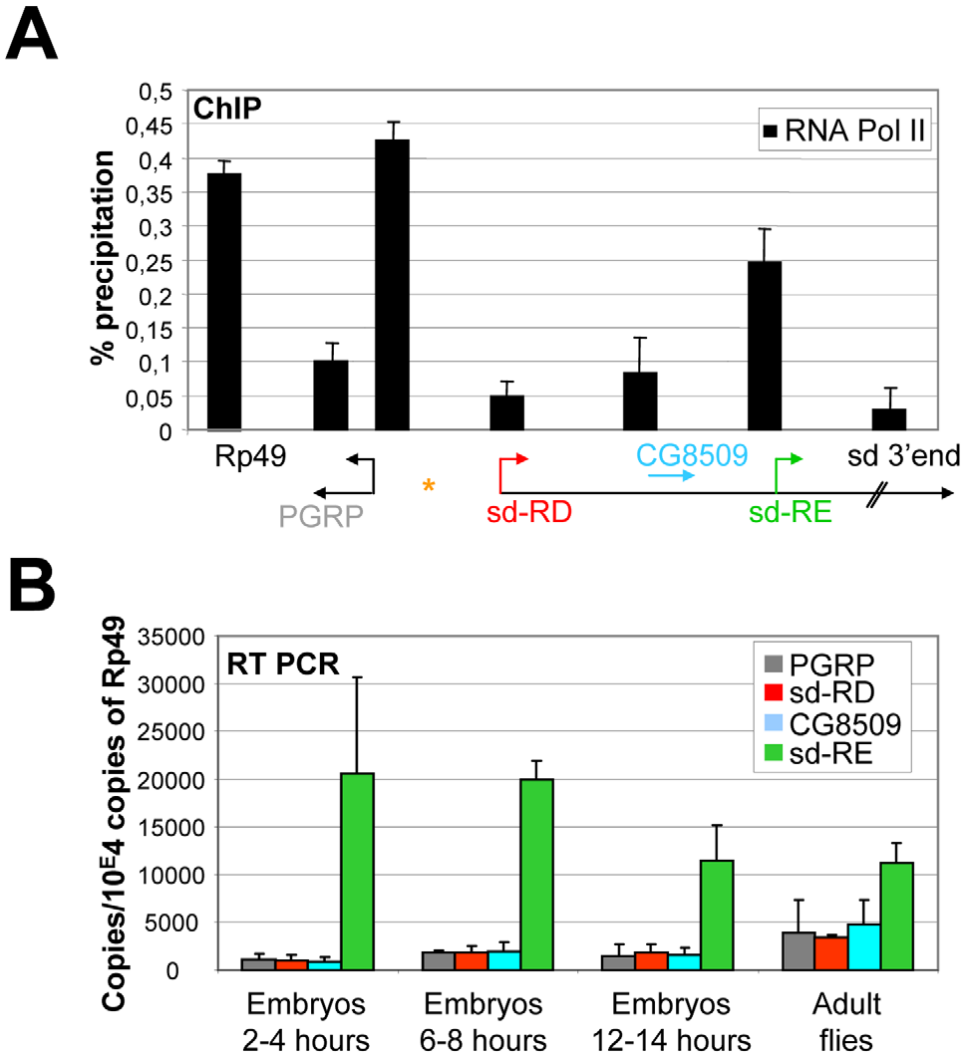
RNA Polymerase II binding and expression of genes at the *sd* gene locus during *Drosophila* development. (A) qChIP analysis on 4–12 hours old wild type embryos using RNA Pol II antibodies. Immunoprecipitated DNA was analysed by quantitative PCR using primers amplifying genomic regions at the *sd* gene locus or the *Rp49* gene. Annotated genes are shown below the graph and are drawn in scale. ChIP signal levels are represented as percentage of input chromatin precipitated for each region. The standard deviation was calculated from at least two independent experiments. Orange asterisk represents the transgene insertion site. (B) qRT PCR analysis of *PGRP*-LE, *sd*-RD, *CG8509* and *sd*-RE gene expression during *Drosophila* development. RNA was extracted from staged embryos at different times after egg laying or from female adult flies. RNA levels were normalized to the housekeeping gene *Rp49*. The standard deviation was calculated from at least two independent experiments. Primer pairs amplifying exon/intron junctions were used to determine levels of nascent transcripts.

To assess the potential role of transcription in demarcating the ectopic H3K27me3 domain, we first determined binding levels of RNA Pol II at the promoter regions of genes flanking the Fab-X transgene insertion site at the embryonic stage. qChIP analysis in wild type (WT) embryos showed that RNA Pol II and H3K4me3 levels correlated strongly at the examined promoter regions (Figure 2A). High levels of RNA Pol II could be detected immediately upstream the transgene insertion site, close to the *PGRP*-LE promoter region, and at the *sd*-RE promoter region, which are also tri-methylated on H3K4. In contrast, only low levels of RNA Pol II were detected at promoter regions of the *sd*-RD transcript and the *CG8509* gene, which are not tri-methylated on H3K4. In addition, low levels of RNA Pol II were observed in the coding region of examined genes.

We next performed qRT-PCR experiments to examine expression of genes at the sd gene locus during *Drosophila* development (Figure 2B). We determined levels of nascent transcripts by using primer pairs amplifying exon/intron junctions of the transcripts. High levels of expression from the *sd*-RE promoter could be detected in early embryogenesis (2–4 hours after egg laying). Expression levels are slightly decreased at later developmental stages but stay elevated throughout fly development. In contrast, no significant transcription can be detected from the *sd*-RD and *CG8509* gene loci in early embryonic development and these genes become moderately activated at later developmental stages. Surprisingly, a similar expression pattern was observed for the *PGRP*-LE gene, whose promoter region is bound by RNA Pol II and marked by H3K4me3 in embryos, but is not detectably transcribed in embryonic development. Together, these results show that promoter regions poised for gene activation by the presence of the RNA Pol II complex, and H3K4me3 demarcate the artificial PcG domain, indicating a potential role of active chromatin components in delimiting these repressive H3K27me3 domains without a need of active transcription *per se*.

### Long-range Interactions between Multiple Copies of the *Fab-7* Element Enhance Binding of PcG Proteins to Chromatin

**Figure 3:**
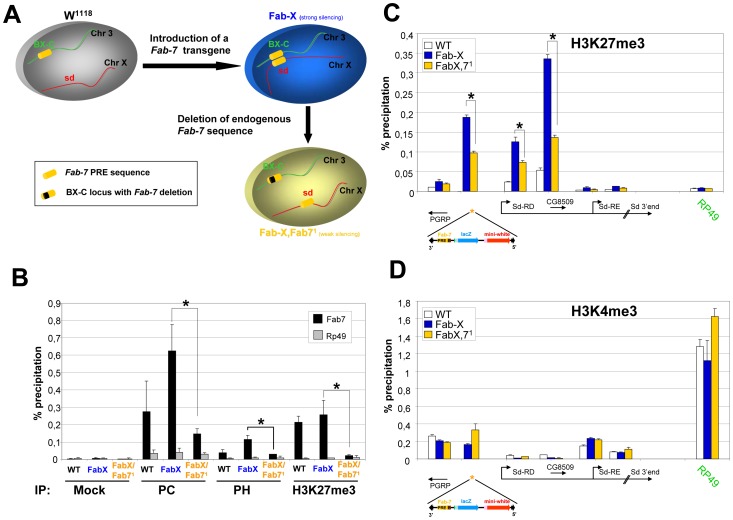
Chromatin state of the *Fab-7* PRE and the *sd* gene locus as a function of *Fab-7* long-range interactions in embryos. (A) Insertion of the a transgene containing a *Fab-7* element (shown as yellow cylinder) at the *scalloped* (*sd*) gene locus leads to pairing with the endogenous *Fab-7* element at the BX-C and increased silencing of the *mini-white* reporter gene (Fab-X). Long range interaction of *Fab-7* elements is dependent on sequence homology: Deletion of the endogenous *Fab-7* sequence (represented as black box), giving rise to the Fab-X, *Fab-7* ^1^ fly line results in loss of pairing and reduced silencing of the *mini-white* reporter gene. (B) qChIP analysis on 4–12 hours old embryos. Fly genotypes and antibodies used for IP are indicated at the bottom of the graph. Immunoprecipitated DNA was analysed by quantitative PCR with the primers amplifying the *Fab-7* element, or the *Rp49* gene. ChIP signal levels are represented as percentage of input chromatin precipitated for each region. The standard deviation was calculated from at least two independent experiments. Note that *Fab-7* primers amplify both, the endogenous *Fab-7* sequence and the transgenic element. (*P<0.05 as calculated from a two-tailed t-test). (C) and (D) ChIP analysis on 4–12 hours old embryos using H3K27me3 antibodies (C) or H3K4me3 antibodies (D). Immunoprecipitated DNA was analysed by quantitative PCR using primers amplifying genomic regions at the *sd* gene locus or the *Rp49* gene. Annotated genes are shown below the graph and are drawn in scale. ChIP signal levels are represented as percentage of input chromatin precipitated for each region. The standard deviation was calculated from two independent experiments. (*P<0.05 as calculated from a two-tailed t-test).

We have previously shown that PcG-mediated repression is enhanced by *trans* interactions of the transgenic *Fab-7* copy with the endogenous *Fab-7* element at the BX-C [28], but the effect of these long range interactions on chromatin structure and composition is unknown. *Fab-7* mediated pairing was shown to be dependent on sequence homology since deletion of the endogenous *Fab-7* copy (giving rise to the Fab-X, *Fab-7* ^1^ line) results in loss of pairing of the BX-C with the transgenic *Fab-7* copy and reduced silencing of the reporter gene present in the construct as well as of the *sd* gene. Figure 3A shows a schematic representation of the Fab-X transgenic system.

We set out to test whether the long-range interaction between the PcG domain induced by the transgene at the *sd* gene locus and the endogenous PcG domain at the BX-C has an impact on PcG binding. Therefore, we compared levels of PcG proteins and of H3K27me3 at the *Fab-7* PRE and at flanking genomic regions of the *sd* gene locus in the presence or absence of *Fab-7* interaction with the BX-C. qChIP experiments in *Drosophila* embryos revealed that the PcG proteins Polycomb (PC) and Polyhomeotic (PH) are detected at the transgenic *Fab-7* element in the absence of the endogenous copy in embryos (see Fab-X;Fab7^1^ line Figure 3B). Importantly, in the presence of the endogenous *Fab-7* element (Fab-X line) we detected significantly increased levels of PcG proteins at the *Fab-7* PRE (Figure 3B) and much increased H3K27me3 levels in the flanking genomic regions of *sd*, up to the *sd-RE* promoter (Figure 3C). In contrast, levels of H3K4me3 at genes bordering the ectopic domain do not change significantly upon insertion of the *Fab-7*-containing transgene (Figure 3D). Similar results were obtained by ChIP analysis of chromatin from adult flies (Figure S3).

In summary, we showed that PcG proteins and their associated histone mark H3K27me3 are recruited by the *Fab-7* element to the transgene and to the *sd* gene locus at a lower level in the absence of the endogenous *Fab-7* element at the BX-C. This indicates that long-range interaction between PcG domains increases the amount or affinity of PcG proteins bound to chromatin, rather than the size of the genomic region covered by them. Importantly, the overall PcG-binding profile at the BX-C locus does not change significantly as a function of long-distance interactions of the *Fab-7* copies (Figure S4A and S4B), suggesting that the large number of PREs already present at the BX-C recruits large amounts of PcG proteins and renders the BX-C chromatin insensitive to the addition of an individual PRE-containing transgene.

### Decreased Transcription of Genes Incorporated into a PcG Domain Correlates with Reduced Binding of RNA Pol II at H3K27me3 Marked Promoters

**Figure 4:**
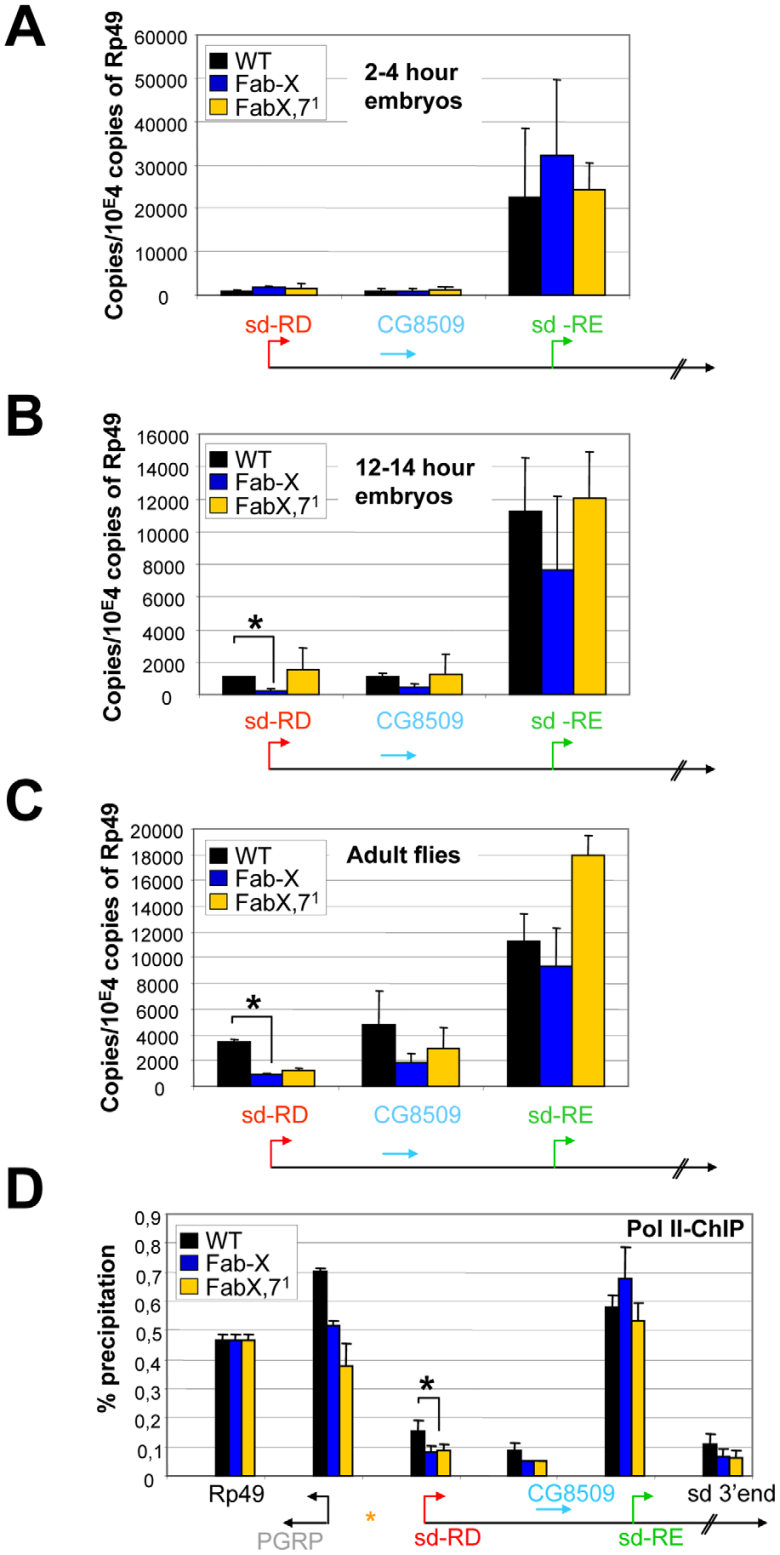
RNA Polymerase II binding and expression of genes at the *sd* gene locus during *Drosophila* development before and after insertion of the *Fab-7* containing transgene. (A–C) qRT PCR analysis of *sd*-RD, *CG8509* and *sd*-RE gene expression during *Drosophila* development in WT, Fab-X and Fab-X,*Fab-7* ^1^ flies. RNA was extracted from staged embryos 2 hours (A) 12 hours (B) after egg laying or from female adult flies (C). RNA levels were normalized to the housekeeping gene *Rp49*. The standard deviation was calculated from at least two independent experiments. Note that primers amplifying the *sd*-RE transcript also amplify the *sd*-RD transcript. (D) qChIP analysis of female adult flies in the indicated fly lines using RNA Pol II antibodies. Immunoprecipitated DNA was analyzed by quantitative PCR using primers amplifying genomic regions at the *sd* gene locus or the *Rp49* gene. Annotated genes are shown below the graph and are drawn to scale. ChIP signal levels are represented as percentage of input chromatin precipitated for each region. The standard deviation was calculated from at least two independent experiments. (*P<0.05 as calculated from a two-tailed t-test).

We next tested how transcription of genes within the ectopic PcG domain is affected as a function of nuclear redistribution leading to increased H3K27me3 levels. Therefore we determined transcription levels of genes at the *sd* gene locus before and after the transgene insertion in the presence (Fab-X) or absence (Fab-X, *Fab-7* ^1^) of *Fab-7* pairing during *Drosophila* development (Figure 4A–C). As shown before, at an early embryonic stage, the *sd*-RD transcript and the *CG8509* gene are not significantly transcribed, whereas the *sd*-RE transcript is highly expressed, and expression is not significantly affected upon insertion of the *Fab-7* transgene (Figure 4A). However, at the end of embryonic development and in adult flies, the two previously inactive genes are induced in wild type (WT) flies, whereas in the Fab-X line transcription levels are significantly lower (Figure 4B and 4C). Importantly, the presence of *Fab-7* in the Fab-X transgene did not completely shut down transcription, but reproducibly decreased the transcriptional output 3-fold in the case of the *sd*-RD transcript and 2 to 3-fold in the case of *CG8509*. This reduced expression can be partially rescued upon deletion of the endogenous *Fab-7* copy (Fab-X, *Fab-7* ^1^ line), which can explain the milder scalloped phenotype observed in Fab-X, *Fab-7* ^1^ flies [28]. Thus, the simple insertion of *Fab-7* cannot efficiently silence genes far away from its integration site. In contrast, long-range interaction of the artificial PcG domain at the *sd* gene locus with the BX-C can decrease transcription significantly. Notably, no major changes in expression levels of more distal genes (located up to 100 kb away from the transgene insertion site) could be detected at the embryonic or adult stage (Figure S5), nor did we detect chromatin changes at these gene regions (data not shown), indicating that the silencing effects mediated by the *Fab-7* transgene are limited to distances of several kilobases from the PRE insertion site.

It has been previously reported that PcG-target genes and RNA Pol II binding were largely exclusive [15] suggesting an effect of PcG proteins on RNA Pol II recruitment to target promoters. On the other hand it has been demonstrated that PcG proteins and basal transcription factors can colocalize at promoter regions [8], and that PcG-mediated silencing can take place at the level of transcriptional elongation [9]. To examine whether RNA Pol II binding at promoter regions of genes becoming incorporated into the artificial domain is affected, we determined the levels of RNA Pol II to chromatin before and after insertion of the *Fab-7* containing transgene. No significant changes in levels of RNA Pol II could be detected after insertion of the transgenic *Fab-7* copy in early embryos (data not shown). However, at the adult stage we observed a 2-fold decrease in RNA Pol II levels at the *sd*-RD and *CG8509* promoter regions in Fab-X versus wild type (WT) flies (Figure 4D). These results indicate that H3K27me3-marked chromatin reduces RNA Pol II promoter occupancy to decrease transcriptional output. We note, however, a similar effect in the Fab-X,7^1^ line, where silencing is less strong, suggesting that part of the silencing may be explained by a slight PcG-dependent reduction in elongation efficiency.

## Discussion

In this study we analyzed the mechanisms impinging on the formation, stability and silencing ability of an ectopically generated PcG domain. Our data show that PcG proteins bind specifically to the transgenic PRE. Importantly, chromatin surrounding the PRE-containing transgene becomes tri-methylated on H3K27, forming a repressive H3K27me3 domain of over ten kb in size. We found that H3K27me3 can spread over boundary elements and silent gene promoters, whereas promoter regions of genes that are either expressed or poised for activation demarcate the PcG domain. We propose that protein complexes associated with active promoter regions are major determinants in blocking H3K27me3 spreading. Furthermore, chromatin analysis of the transgenic Fab-X system showed that long-range interactions of Fab-7 elements increase the efficiency of PcG binding, resulting in enhanced gene silencing and decreased RNA Pol II affinity to promoter regions. To our knowledge this is the first report demonstrating the effect of long-range interactions of PREs on chromatin structure.

### PRE-dependent Generation of PcG Domains

The insertion of the *Fab-7* PRE at genomic loci that usually have low PcG-binding affinity is sufficient for recruitment of PcG proteins and for marking of the transgene as well as the surrounding chromosome region with H3K27me3. Is this mark deposited by spreading of PcG complexes or by PRE-bound complexes looping out to reach neighboring chromatin regions? Interestingly, we did neither observe homogeneous levels of H3K27me3, nor did we see a gradual decrease of H3K27me3 levels from the PRE outwards. Instead, peaks and valleys appear in the profile. This argues against a linear spreading of PcG proteins along the chromatin fiber. A more likely scenario is that PcG complexes bound at PREs may loop out and contact downstream chromatin regions. Indeed, we have shown recently that a transgenic PRE can contact a downstream promoter via chromatin looping [34]. Therefore it is tempting to speculate that the PRE-bound methyltransferase activity may modify nucleosomes of the contacted regions, with higher levels of methylation arising at regions that maintain the contacts with the PRE for longer time periods. Thus, dynamic chromatin contacts resulting in histone methylation may produce the apparent spreading of H3K27me3 observed in ChIP-chip (reviewed in [35]).

Importantly, we detect weak signals of PC and H3K27me3 at the *sd* gene locus in wild type flies (Figure 1B and 1D or Figure 3B and 3C). Thus the *sd* locus seems to have intrinsic weak affinity for PcG proteins and insertion of the *Fab-7* containing transgene may increase PcG affinity to this locus. One might hypothesize that many regions with weak intrinsic affinity for PcG proteins exist in the *Drosophila* genome, which on their own are not sufficient to stably recruit PcG proteins to chromatin. However, upon insertion of a PRE close to these sites, the affinity to these weak sites could increase, resulting in enhanced PcG binding.

### Confinement of H3K27me3 Domains

On a genome wide scale it has been observed that many H3K27me3 domain borders correlate with the presence of known insulator proteins, in particular CP190/CTCF, GAF and Mod(mdg4) [21]–[23]. In addition to insulator proteins, promoter regions of actively transcribed genes and the presence of RNA Pol II is a significant feature of H3K27me3 borders [18], [22], [24]. Interestingly we found that the boundary sequence present in the *Fab-7* element did not interfere with the propagation of the H3K27me3 mark. Moreover, comparison with ChIP-on-chip data of known insulator proteins [21] argues against a one to one relation between the presence of these chromatin insulators and the stop of spreading of H3K27me3 from the *Fab-7* containing transgenes. However, we cannot exclude the formal possibility that other endogenous boundary elements located at the *sd* gene locus could account for the stop of spreading of the repressive histone mark from the transgenic PRE.

We found that H3K27me3 can spread from a transgenic PRE over gene promoter regions where H3K4me3 and RNA Pol II are absent, whereas promoters marked by RNA Pol II and H3K4me3 demarcate the borders of the PcG domain. Likewise, many endogenous PcG domains are sharply bordered by gene promoter regions associated with active histone marks [18], [22], [24], strongly suggesting that components associated to active chromatin may play a major function in blocking the spreading of PcG domains. The fact that no transcripts can be detected in the upstream region of the transgene insertion site argues against the requirement of the transcription process itself for the boundary function.

What is the molecular mechanism at the basis of the block? One possibility is that the active mark H3K4me3 directly interferes with the deposition of a methyl mark on H3K27. H3K4me3 has been shown to inhibit methyltransferase activity catalyzed by PRC2 activity in an allosteric fashion when nucleosomes are methylated on lysine 4 in a symmetric manner [36], [37]. However, the fact that H3K27me3 domain borders and adjacent H3K4me3 peaks are slightly offset is arguing against a direct interplay of the activating and repressing histone marks. On the other hand, effector proteins bound at the H3K4me3 mark could impede the substrate for the H3K27 methyltransferase. A second possibility is that the transcriptional pre-initiation complex or co activators bound at promoter regions might act as physical barriers blocking the spreading of H3K27me3. Moreover, chromatin insulator components are strongly associated to promoters, suggesting that promoters may be frequently endowed with an intrinsic insulator or barrier function and those insulator proteins may be involved in Polycomb domain definition by contributing to promoter function. One particularly good candidate protein for the insulator function at the *sd* gene locus is the chromatin regulator GAF. GAF has been shown to counteract heterochromatin spreading by directing histone H3 replacement by the H3.3 variant [38]. In addition, GAF can act as a bridging factor that facilitates communication of regulatory sequences and has been suggested to confer insulator function via trapping of distal enhancers. The fact that we did not observe an increased scalloped mutant phenotype (corresponding to an increased spreading of H3K27me3 mark) in a heterozygous GAF mutant background argues against an essential role of GAF as boundary factor. However, we cannot exclude the possibility that reduction of the GAF dosage by half was not sufficient to inhibit its boundary function. Finally, promoter-bound complexes may recruit H3K27-specific histone demethylases, which may actively remove the H3K27 methyl marks in the vicinity of their binding sites. The histone demethylase UTX has been shown to associate with elongating RNA Pol II in *Drosophila* [39] and human UTX associates with Trithorax group (TrxG**)** family members [40]. Trithorax (TRX) binding is highly correlated with H3K4me3-marked promoter regions [18], and we found TRX to be bound at the *sd* promoter region that is tri-methylated at H3K4 and interfere with the spreading of the H3K27me3 mark (data not shown). Therefore, insulator proteins, transcription factors and cofactors, the TrxG machinery and RNA polymerase II are candidate to delimit PcG domains. Whether they block spreading collectively or whether one of the proteins suffices to fulfill this function remains to be studied.

### The Effect of *Trans* Interaction of PREs on the Chromatin Structure of *Fab-7* Sequences during *Drosophila* Development

PcG proteins have a speckled distribution within the nucleus [29]. The number of these so-called PcG bodies [41]–[43] progressively decreases during development and is significantly smaller then the number of genomic binding sites of PcG proteins determined by ChIP-on-chip assays [29]. Analysis of the location of PcG-target genes demonstrated that PcG bodies correspond to physical sites of gene silencing [30], and multiple copies of the *Fab-7* PRE can associate in the nucleus to enhance PcG-mediated silencing [28]. Finally, many endogenous PcG target genes associate in the nuclei and this association can increase the silencing, at least in the case of Hox genes [27], [30]. Together, these previous observations showed that clustering of PRE-containing regions within subnuclear silencing compartments is relevant for gene regulation during development. Indeed, we found that binding of PcG proteins at the *sd* gene locus on the X chromosome is stronger when the endogenous *Fab-7* element is present at the BX-C in chromosome 3, showing that long-distance interactions between these loci can reinforce silencing by stabilizing the association of PcG proteins with chromatin. However, a single *Fab-7* transgene is sufficient for the recruitment of PcG proteins and its associated histone mark H3K27me3 even in the absence of the endogenous copy, although binding levels of PcG proteins are significantly lower and H3K27me3 is just above background. Strongly increased H3K27me3 levels at the *Fab-7*-containing transgenic locus in the Fab-X line may be due to targeting of the transgene to a large PcG body and subsequent communication with the BX-C locus [29]. In this configuration, the large amounts of PcG proteins that are present in the three-dimensional surroundings may be more easily recruited by the PRE. This suggests that the chromosomal environment of a PRE has an impact on PcG complex targeting.

A previous report demonstrated physical interaction of multiple PREs at the BX-C locus [26] and these interactions in *cis* might be essential for efficient recruitment of PcG proteins. In line with this hypothesis, ChIP-chip analysis of PcG proteins in *Drosophila* embryos [18] shows a significant correlation between the intensity of H3K27me3 signals and the number of PREs in a PcG domain (Figure S6), suggesting that PRE interactions may stabilize PcG-dependent silencing throughout the genome. This tendency towards clustering does not obligate individual PREs to be repressed. For instance, the endogenous *Fab-7* sequence at the BX-C is not bound by PcG proteins at the adult stage, although the other PREs of the locus stay efficiently bound. This dynamic PcG behavior, which was previously observed for other PREs [14], [44], [45], suggests that global locus control by PcG proteins is compatible with stage-specific PRE-regulation.

In summary, *cis*-regulation imposed on the surrounding DNA by individual PREs, global locus regulation relying on higher-order chromatin folding with the contribution of multiple PREs from the same locus and *trans*-regulation involving three-dimensional contacts among PREs located at large distances might contribute to impart to each PcG target locus its specific chromatin state leading to its precise regulatory output.

## Materials and Methods

### Fly Lines

Flies were raised in standard corn meal yeast extract medium. The Oregon-R w^1118^ line (referred to as wild type) was obtained from R. Paro (ZMBH, University of Heidelberg, Germany). Transgenic lines used in this study have been previously described in [28]. In the Fab-X and Fab-X, *Fab-7* ^1^ lines the Fab-7 containing transgene is inserted at position 15698606 of chromosome X, 1.648 kb downstream of the *PGRP-LE* gene and 1.670 kb upstream of the *sd*-RD transcript (flybase release FB2008_07). For all experiments including the Fab-X and Fab-X, *Fab-7* ^1^ lines, flies were grown at 28,5°C since PcG-mediated silencing was stronger at higher temperatures.

### Chromatin Immunoprecipitation (ChIP) and ChIP-on-chip Experiments on Whole *Drosophila* Embryos or Adult Flies

ChIP of whole embryos or homozygous adult flies was essentially performed as previously described [14]. Briefly, cross linking was performed for 15 min in the presence of 1.8% formaldehyde during tissue homogenization. Chromatin extracts of embryos were sonicated using a Bioruptor (Diagenode) for 15 min (settings 30 sec on, 30 sec off, high power). Chromatin extracts of adult flies were sonicated for 20 min (settings 30 sec on, 30 sec off, high power). Sheared chromatin had an average length of 500 to 1000 bp. Antibodies used for IP were diluted 1∶100 for IP. PC, PH antibodies are described in [18], H3K4me3 (#07–473), H3K27me3 (#07–449) and RNA Pol II (#05–623) antibodies were from UPSTATE Biotechnology.

For quantitative ChIP, after immunoprecipitation and DNA purification, enrichment of specific DNA fragments was analyzed by real-time PCR, using the Roche Light Cycler equipment and accessories as described in [19]. Data are expressed as the percentage of input chromatin precipitated for each region examined. As a negative control, the *Rp49* was used in the PCR experiments.

For ChIP-on-chip assays precipitated DNA was amplified by ligation mediated PCR (LM PCR). To verify the linear response of the LM PCR, amplified DNA was tested by Southern blot analysis using genomic DNA fragments containing known PcG binding sites [46]. 4 µg of each amplified sample was sent to Nimblegen Systems Inc. for hybridization on the microarrays.

### Microarray Details

A 385K tiling microarray representing the euchromatic, non-repetitive regions of *Drosophila melanogaster* genome sequence (Flybase release 4.3) with an average oligonucleotide length of 50 bp and an average inter-oligo spacing of 130 bp from Nimblegen Systems Inc. (GEO accession: GPL3353) were used to hybridize embryo ChIP samples (H3K4me3 and H3K27me3) and adult ChIP sample (H3K4me3). Adult ChIP sample (H3K27me3) was hybridized on a 385K genome tiling array provided by NimbleGen (Design Name: DMEL ChIP Set3) with an oligonucleotide length of 50 bp and a median probe spacing of 97 bp. Specific (Cy5) and Mock IP (Cy3) signal intensities as quantified and provided by Nimblegen were converted to log2 ratio and normalized using loess normalization [47]. Array data can be accessed on: http://cav-ouranos.igh.cnrs.fr/data/NIMBLEGEN_DATA_Schuttengruber_Cavalli.rar

A single replicate was performed of WT and Fab-X lines in embryos and adults. In these cases, spreading of repressive histone marks from the PRE-containing transgene and formation of the artificial PcG domains was confirmed by ChIP experiments analyzed by quantitative PCR (see Figure 1). Thus in total at least 3 independent ChIP experiments have been performed to analyze the formation of the artificial PcG domains. In addition the accuracy of the ChIP-on-chip profiles have been validated by comparing them with our previously published profiles from 4–12 hours embryos [18].

### RNA Purification and RT-qPCR Analysis

RNA isolation of whole *Drosophila* embryos or adult flies was done using Trizol Reagent according to the manufacturer’s instructions (Sigma). RT PCR was performed using Superscript III First Strand Synthesis Kit from Invitrogen following the manufacturer’s instructions. Reverse transcription was primed using hexamer primers. qPCR analysis was done as described for ChIP experiments. The copy number for each investigated gene was normalized to a housekeeping gene (*Rp49*). To detect nascent transcripts, at least one primer of the amplicon is located within an intron of the investigated gene. Primer sequences are listed in Table S1.

### H3K27me3 Domain Intensity

Genome wide H3K27me3 domain data were derived from [18]. The average intensity in a domain was calculated by dividing the area covered by the binding intensity by the size of the domain. A correlation was drawn between the numbers of PH peaks to the average binding intensity seen in a domain. Out of 439 PH peaks, which showed significant levels of PC binding, 417 were localised in H3K27me3 domains and were taken for this analysis. Two domains in chromosome 3R of HOX clusters spanning 2649952–2874886 bp (14 PH peaks) and 12482959–12811306 bp (30 PH peaks) were excluded from the analysis as they were considered as outliers with large number of PH peaks.

## Supporting Information

Figure S1 **Chromatin state at the** ***mini-white*** **gene of the** ***Fab-7*** **containing transgene.** (A and B) ChIP-on-chip analysis of the *mini white* gene from embryos (A) or adult flies (B) in the indicated fly lines. Although the graph displays the *white* gene as it is annotated in the endogenous genome, the fly lines used in this study carry the *w^1118^* mutation, which deletes almost all DNA sequences at the *white* locus. Thus, the ChIP on chip signal corresponding to *white* comes from the transgene. Fold changes between the specific IP and mock IP are plotted on the Y axis. On the X axis, genomic coordinates and *white* gene are indicated. P indicates the white promoter. CDS marks the white coding region. I-1 indicates the first intron of the endogenous white gene. Note that in the *mini-white* gene of the transgene the first large intron is deleted and therefore no significant ChIP-on-chip signal is detected. Notably, the *white* gene is not expressed during early development and therefore does not interfere with the spreading of the H3K27me3 mark. The weak H3K4me3 signals associated with the white promoter region might represent a subset of cells, where *the mini-white* gene escapes PRE-mediated silencing and gets activated, accounting for the variegated eye phenotype observed in this fly line.
(PDF)

Figure S2 **Distribution of chromatin insulator binding sites at the sd gene locus.** Annotated genes at the sd gene locus (X chromosome position 15628000 to 15668000) are shown on the top. Orange asterisk marks the transgene insertion site. Below, binding sites for the indicated insulator protein are shown [21].
(PDF)

Figure S3 **Chromatin state of the** ***Fab-7*** **PRE and the** ***sd*** **gene locus as a function of** ***Fab-7*** **long-range interactions at the adult stage.** (A) ChIP analysis on female adult flies. Fly genotypes and antibodies used for IP are indicated at the top of the panels. Immunoprecipitated DNA was analysed by semi quantitative PCR with primers amplifying the *Fab-7* element (*Fab-7*), the *white* promoter region (mini-white) or the *Rp49* gene (Rp49). Note that *Fab-7* primers amplify both the endogenous *Fab-7* sequence and the transgenic element. (B) ChIP analysis on female adult flies using H3K27me3 or H3K4me3 antibodies. Immunoprecipitated DNA was analysed by semi quantitative PCR using primers amplifying genomic regions at the *sd* gene locus or the *Rp49* gene. Position of PCR fragments is indicated at the bottom of the figure. Note that the “PRE 5′end” amplicon only amplifies the transgenic Fab-7 element.
(PDF)

Figure S4 **Chromatin state of the bithorax complex (BX-C).** (A) ChIP-on-chip analysis of the BX-C in female adult flies of the indicated fly lines using PC, PH and H3K27me3 antibodies. Fold changes between specific IP the and mock IP are plotted on the Y axis. On the X axis, genomic coordinates and Hox genes are indicated. The *Fab-7* element is indicated by the blue bar. The *bx*, *bxd* and *mcp* PREs are represented by blue asterisks. Note that in WT flies PRC1 components (PC and PH) are not significantly bound at the *Fab-7* sequence, while PC and to a very weak extent PH proteins are bound to the transgenic *Fab-7* copy in the Fab-X, *Fab-7* ^1^ line. In contrast, strong binding of both PRC1 components to *Fab-7* element is observed in the Fab-X line. H3K27me3 levels are lower at the transgenic *Fab-7* copy compared to the endogenous sequences, but do not synergize as a function of *Fab-7* pairing. (B) ChIP-on-chip analysis of BX-C locus in 4–12 hour old embryos of the indicated fly lines using PC, PH and H3K27me3 antibodies. Fold changes between specific IP the and mock IP are plotted on the Y axis. On the X axis, genomic coordinates and Hox genes are indicated. The *Fab-7* element is indicated by the blue bar. The *bx*, *bxd* and *mcp* PREs are represented by blue asterisks.
(PDF)

Figure S5 **Gene expression at the** ***sd*** **gene locus after insertion of the** ***Fab-7*** **containing transgene.** (A) Map of the *sd* gene locus on chromosome X. Orange asterisk indicates the transgene insertion site. (B–C) RT PCR analysis of genes up and downstream the transgene insertion site in embryos (B) or adult flies (C). RNA was extracted from 4–12 hours old embryos or from female adult flies. RNA levels were normalized to the housekeeping gene *Rp49*.
(PDF)

Figure S6 **The number of PREs within a PcG domain correlates with the intensity of H3K27me3.** (A) An example of a domain with lower H3K27me3 intensity (few PH peaks). (B) An example of a domain with higher H3K27me3 intensity (large number of PH peaks). The plots show the ratio (fold change) of specific IP versus mock IP along parts of chromosome 3L and 2R. Significantly bound regions (p-value <1E-04) are indicated in red. (C) Correlation between number of PH peaks and the average intensity of H3K27me3 domains. For calculating the average intensity per domain the area covered by a domain was divided by the size of the domain in bps. Pearson’s correlation co-efficient (CC) was calculated using the R package.
(PDF)

Table S1 **Sequences of primers used in the study.**
(PDF)
