# Supplementary material for: Polycomb Domain Formation Depends on Short and Long Distance Regulatory Cues
Source: PLoS One. 2013 Feb 20;8(2):e56531. doi: 10.1371/journal.pone.0056531 (PMC3577894; doi:10.1371/journal.pone.0056531)
Supplement: Figure S1 — Chromatin state at the mini-white gene of the Fab-7 containing transgene. (A and B) ChIP-on-chip analysis of the mini white gene from embryos (A) or adult flies (B) in the indicated fly lines. Although the graph displays the white gene as it is annotated in the endogenous genome, the fly lines used in this study carry the w1118 mutation, which deletes almost all DNA sequences at the white locus. Thus, the ChIP on chip signal corresponding to white comes from the transgene. Fold changes between the specific IP and mock IP are plotted on the Y axis. On the X axis, genomic coordinates and white gene are indicated. P indicates the white promoter. CDS marks the white coding region. I-1 indicates the first intron of the endogenous white gene. Note that in the mini-white gene of the transgene the first large intron is deleted and therefore no significant ChIP-on-chip signal is detected. Notably, the white gene is not expressed during early development and therefore does not interfere with the spreading of the H3K27me3 mark. The weak H3K4me3 signals associated with the white promoter region might represent a subset of cells, where the mini-white gene escapes PRE-mediated silencing and gets activated, accounting for the variegated eye phenotype observed in this fly line. (PDF) [file pone.0056531.s001.pdf]

A

PcG binding at the *mini-white* gene in Embryos

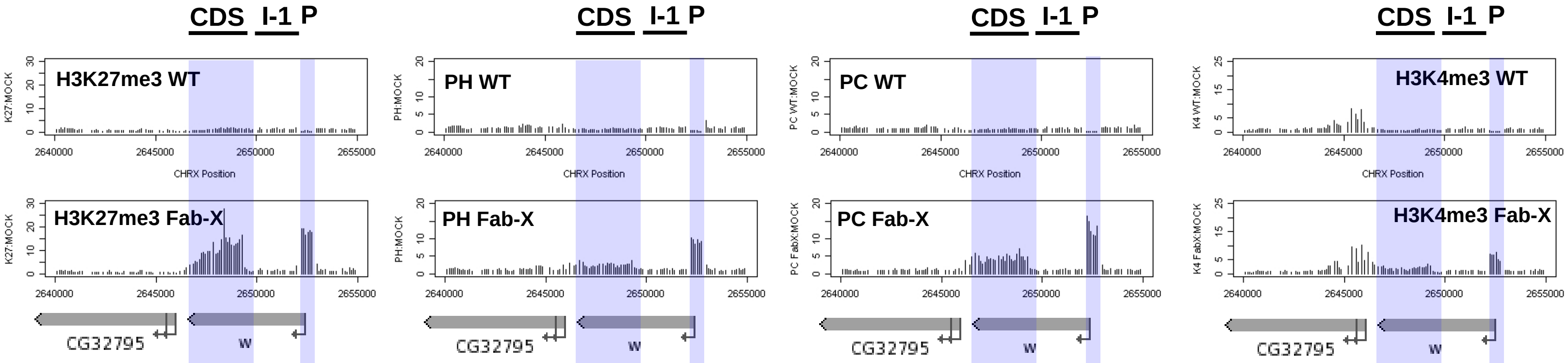

B

PcG binding at the *mini-white* gene in Adult Flies

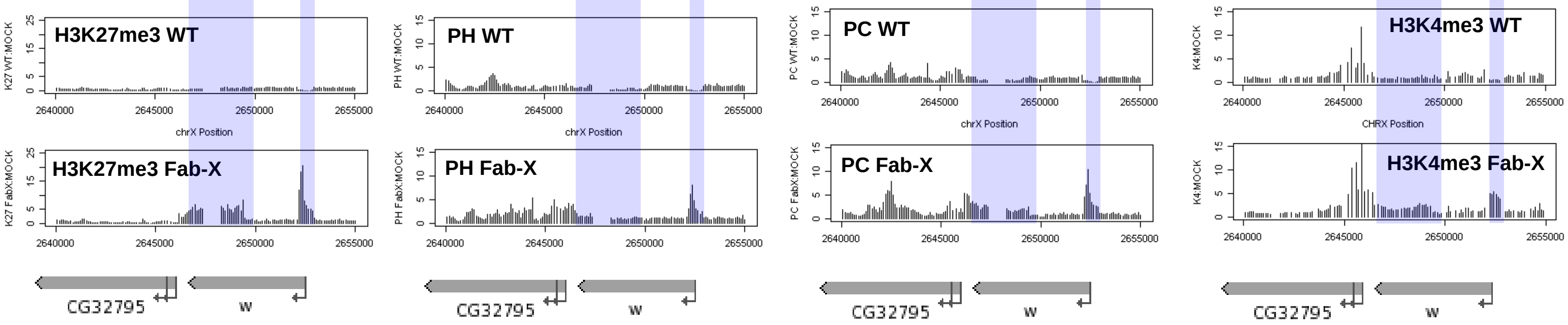

Figure S1
