# Supplementary figures and images for: Polycomb Domain Formation Depends on Short and Long Distance Regulatory Cues
Source: PLoS One. 2013 Feb 20;8(2):e56531. doi: 10.1371/journal.pone.0056531 (PMC3577894; doi:10.1371/journal.pone.0056531)

# ChIP analysis of female adult flies

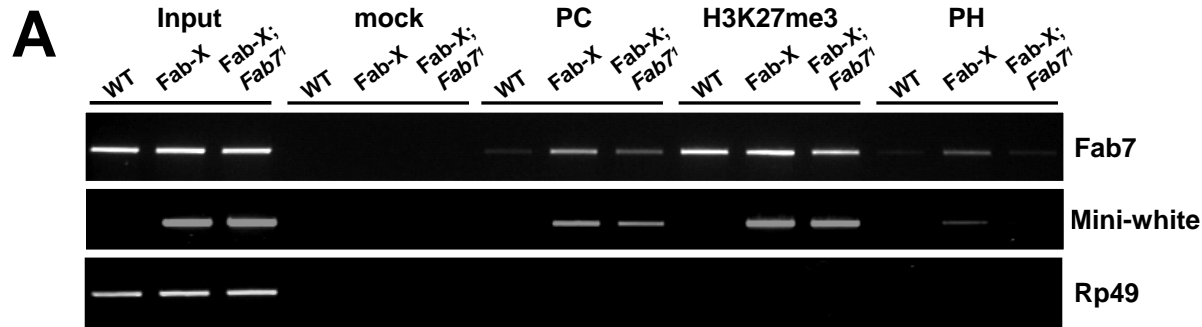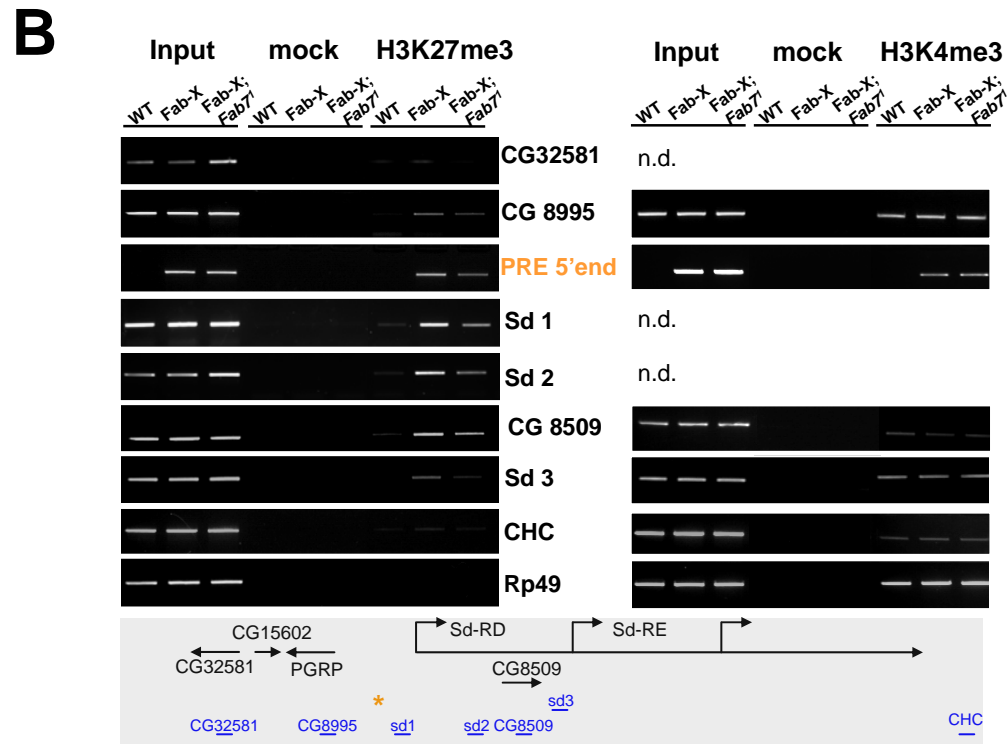

**Figure S3**

Supplement: Figure S3 — Chromatin state of the Fab-7 PRE and the sd gene locus as a function of Fab-7 long-range interactions at the adult stage. (A) ChIP analysis on female adult flies. Fly genotypes and antibodies used for IP are indicated at the top of the panels. Immunoprecipitated DNA was analysed by semi quantitative PCR with primers amplifying the Fab-7 element (Fab-7), the white promoter region (mini-white) or the Rp49 gene (Rp49). Note that Fab-7 primers amplify both the endogenous Fab-7 sequence and the transgenic element. (B) ChIP analysis on female adult flies using H3K27me3 or H3K4me3 antibodies. Immunoprecipitated DNA was analysed by semi quantitative PCR using primers amplifying genomic regions at the sd gene locus or the Rp49 gene. Position of PCR fragments is indicated at the bottom of the figure. Note that the “PRE 5′end” amplicon only amplifies the transgenic Fab-7 element. (PDF) [file pone.0056531.s003.pdf]

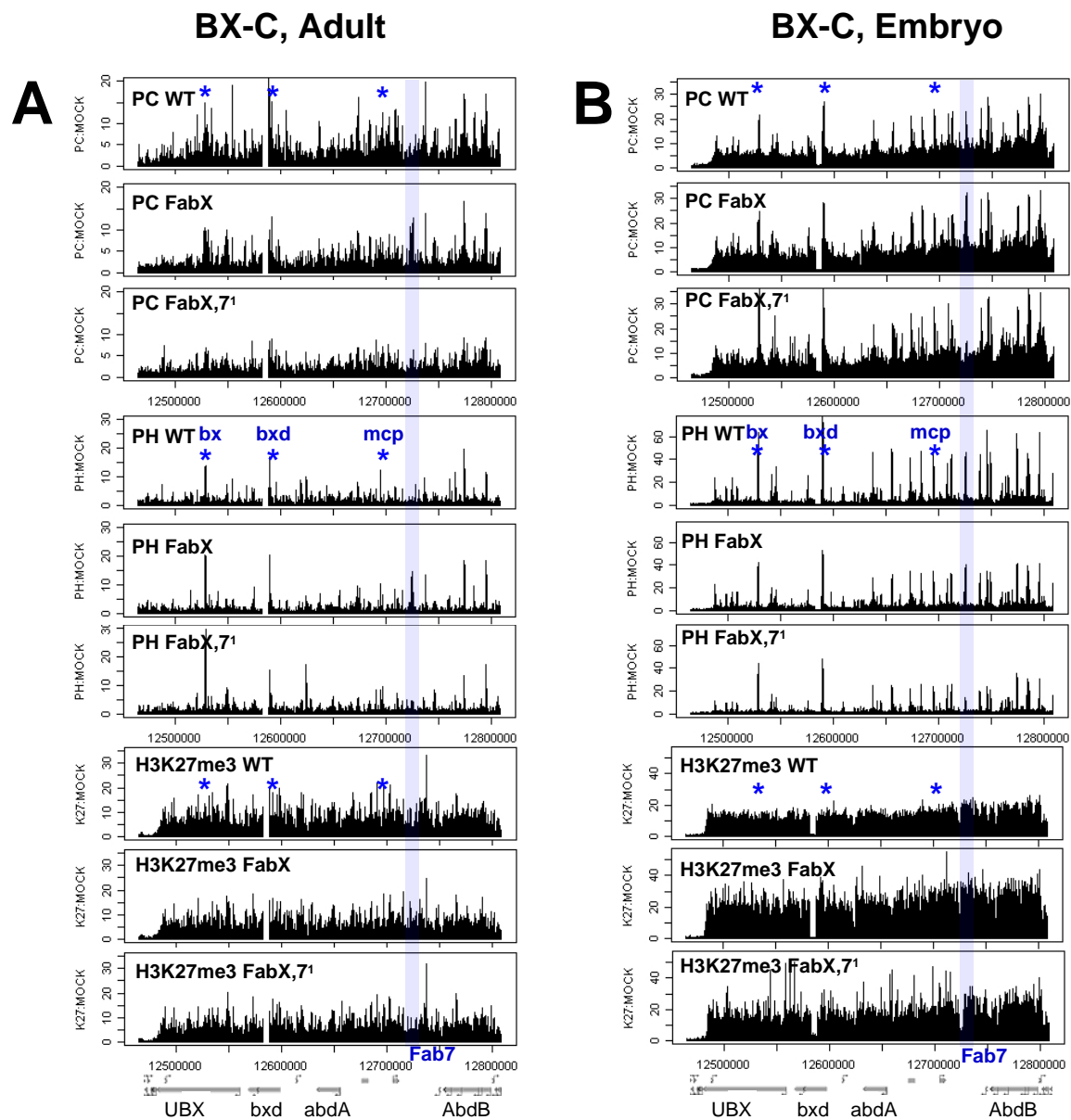

**Figure S4**

Supplement: Figure S4 — Chromatin state of the bithorax complex (BX-C). (A) ChIP-on-chip analysis of the BX-C in female adult flies of the indicated fly lines using PC, PH and H3K27me3 antibodies. Fold changes between specific IP the and mock IP are plotted on the Y axis. On the X axis, genomic coordinates and Hox genes are indicated. The Fab-7 element is indicated by the blue bar. The bx, bxd and mcp PREs are represented by blue asterisks. Note that in WT flies PRC1 components (PC and PH) are not significantly bound at the Fab-7 sequence, while PC and to a very weak extent PH proteins are bound to the transgenic Fab-7 copy in the Fab-X, Fab-7 1 line. In contrast, strong binding of both PRC1 components to Fab-7 element is observed in the Fab-X line. H3K27me3 levels are lower at the transgenic Fab-7 copy compared to the endogenous sequences, but do not synergize as a function of Fab-7 pairing. (B) ChIP-on-chip analysis of BX-C locus in 4–12 hour old embryos of the indicated fly lines using PC, PH and H3K27me3 antibodies. Fold changes between specific IP the and mock IP are plotted on the Y axis. On the X axis, genomic coordinates and Hox genes are indicated. The Fab-7 element is indicated by the blue bar. The bx, bxd and mcp PREs are represented by blue asterisks. (PDF) [file pone.0056531.s004.pdf]

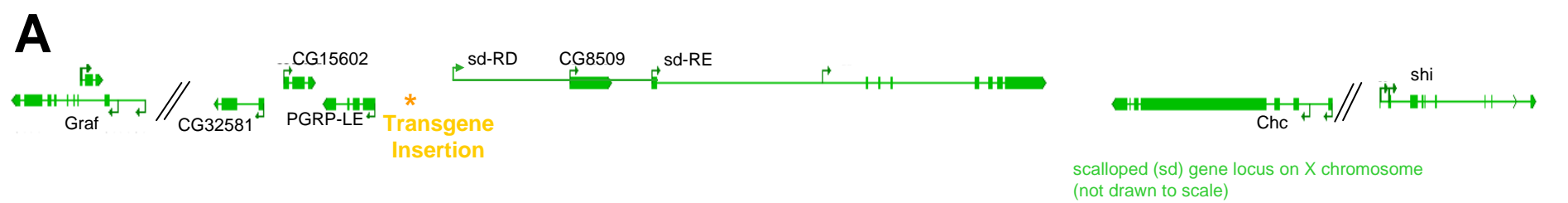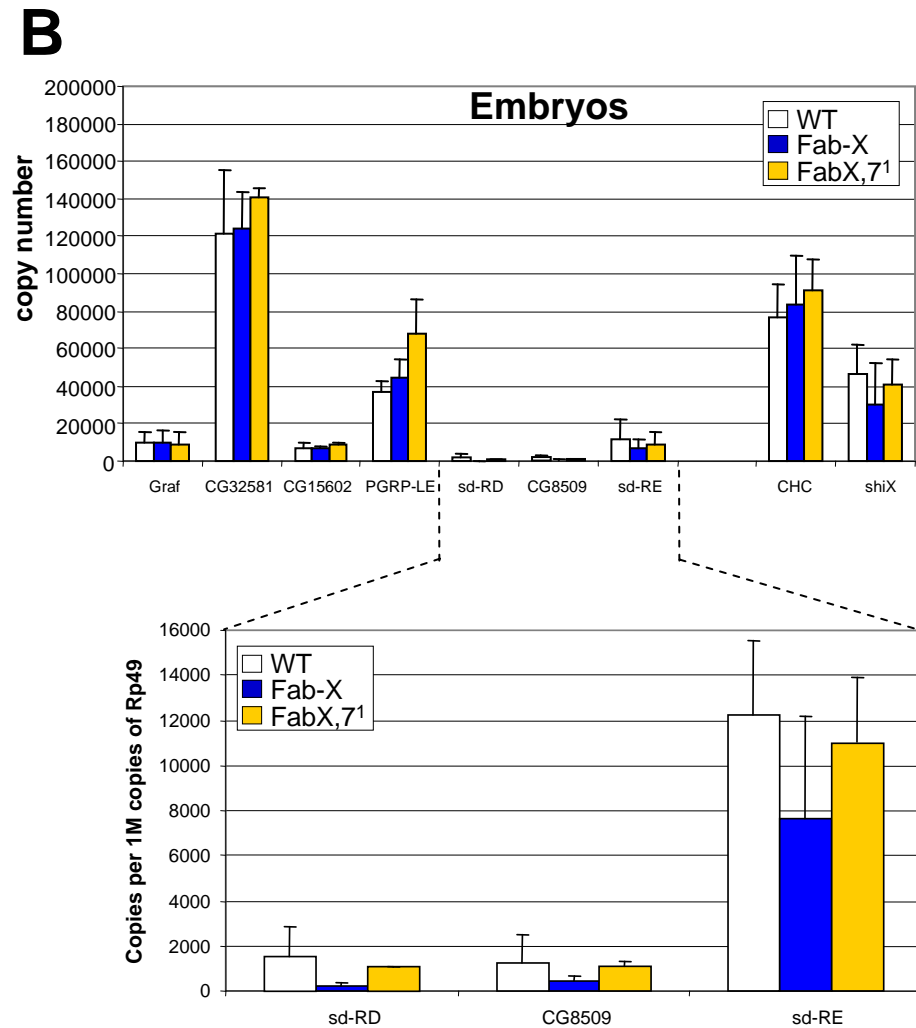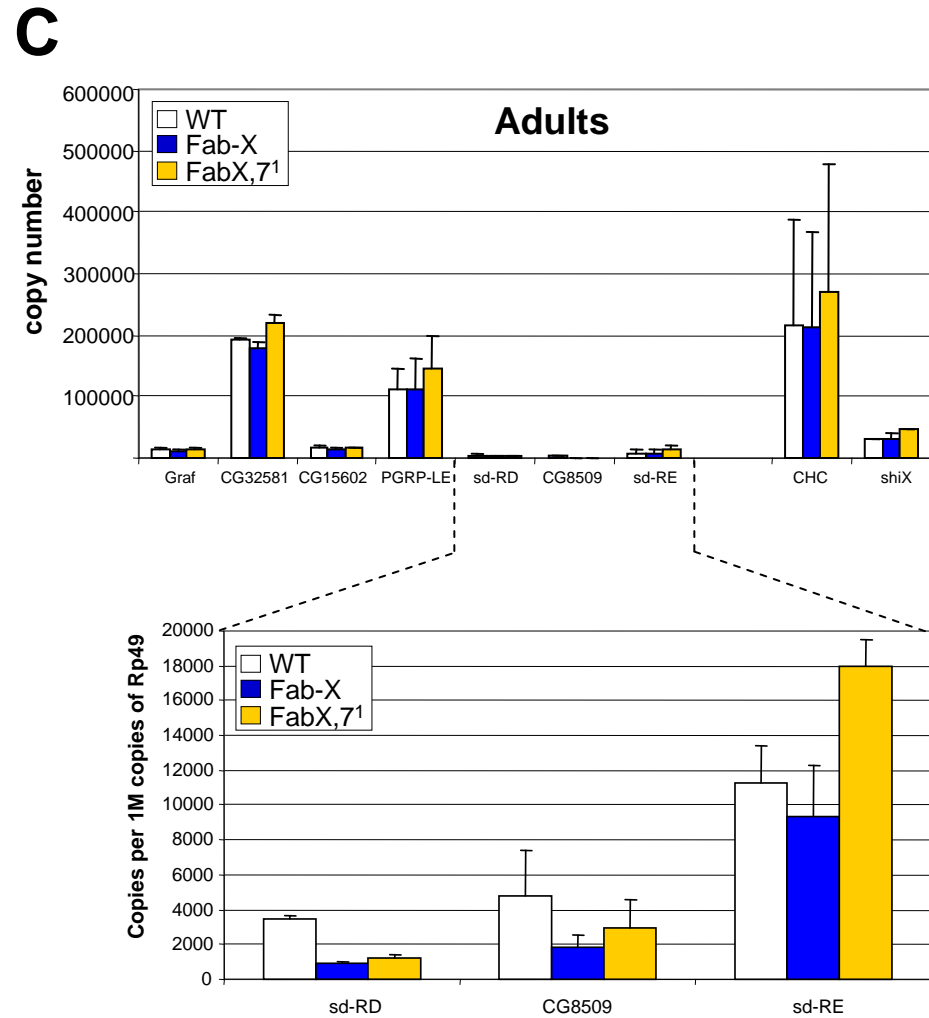

**Figure S5**

Supplement: Figure S5 — Gene expression at the sd gene locus after insertion of the Fab-7 containing transgene. (A) Map of the sd gene locus on chromosome X. Orange asterisk indicates the transgene insertion site. (B–C) RT PCR analysis of genes up and downstream the transgene insertion site in embryos (B) or adult flies (C). RNA was extracted from 4–12 hours old embryos or from female adult flies. RNA levels were normalized to the housekeeping gene Rp49. (PDF) [file pone.0056531.s005.pdf]

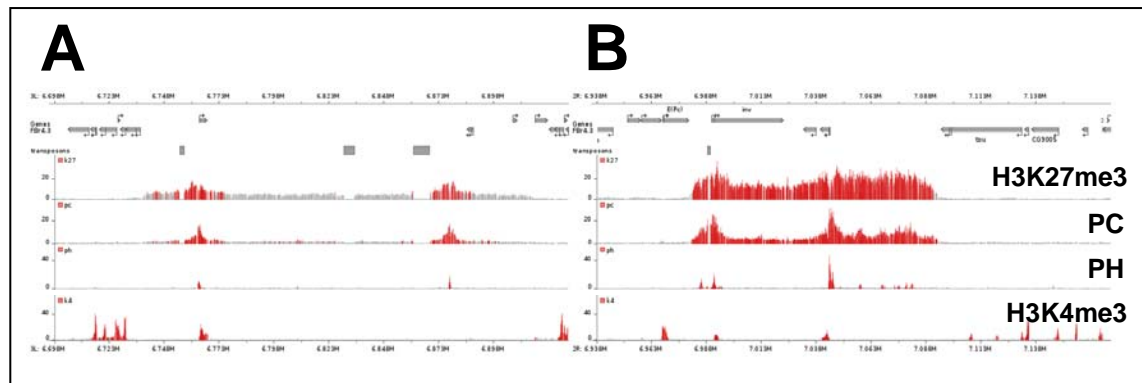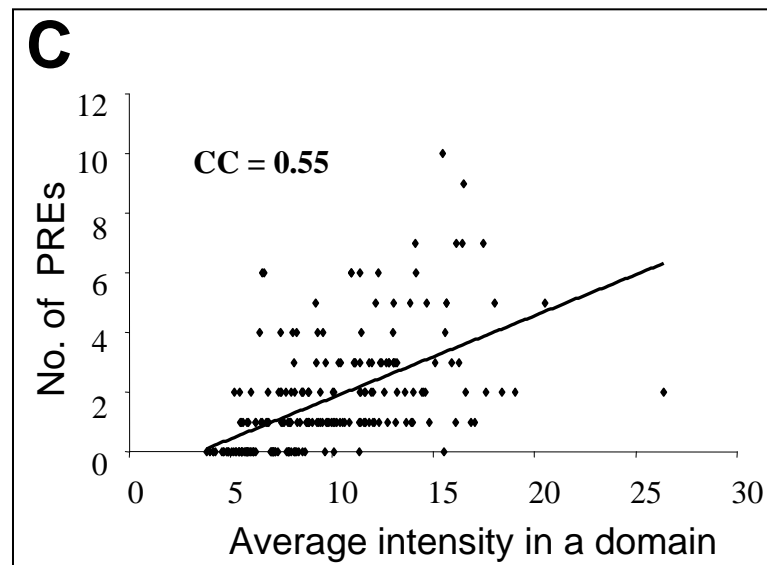

**Figure S6**

Supplement: Figure S6 — The number of PREs within a PcG domain correlates with the intensity of H3K27me3. (A) An example of a domain with lower H3K27me3 intensity (few PH peaks). (B) An example of a domain with higher H3K27me3 intensity (large number of PH peaks). The plots show the ratio (fold change) of specific IP versus mock IP along parts of chromosome 3L and 2R. Significantly bound regions (p-value <1E-04) are indicated in red. (C) Correlation between number of PH peaks and the average intensity of H3K27me3 domains. For calculating the average intensity per domain the area covered by a domain was divided by the size of the domain in bps. Pearson’s correlation co-efficient (CC) was calculated using the R package. (PDF) [file pone.0056531.s006.pdf]
