# Supplementary material for: Polycomb Domain Formation Depends on Short and Long Distance Regulatory Cues
Source: PLoS One. 2013 Feb 20;8(2):e56531. doi: 10.1371/journal.pone.0056531 (PMC3577894; doi:10.1371/journal.pone.0056531)
Supplement: Table S1 — Sequences of primers used in the study. (PDF) [file pone.0056531.s007.pdf]

Table S1. Details of primers used in the study

	Primer name	Sequence (5' to 3')
**Figure 2**	PGRP CDS forward	CCTGGTGAATGATAGCTTACTCTG
	PGRP CDS reverse	CTTACTCAAAACCGAAGAGATCG
	PGRP Prom forward	TGAGCTTTCCAACACTCTTGC
	PGRP Prom reverse	GGTTTTGGTGGTTTATCTGAG
	sd-RD Prom forward	TTCTTCGATCCCTGCAAGGTCC
	sd-RD Prom reverse	GGAGAGCTGTGCGGATTGATTG
	CG8509 Prom forward	TGTCCAATTGCTCCGGGCATC
	CG8509 Prom reverse	ATATGCCTGCGAGCGAACCCA
	sd-RE Prom forward	CTGCTGCTCTTGCTGTATTTTGC
	sd-RE Prom reverse	GAAGCGAGAGAAACTCATCGAC
	sd 3'end forward	CGTAACCAGCCAGTAAGTATTG
	sd 3'end reverse	CCTTCTCCACCACTTGCTTG
**Figure 3C-D**	PGRP forward	CCTGGTGAATGATAGCTTACTCTG
	PGRP reverse	CTTACTCAAAACCGAAGAGATCG
	Transgene insertion forward	CTTGCGTCTGTCTGTGTTATTTCG
	Transgene insertion reverse	CCTCCACTACGGGTAAATTCC
	sd-RD forward	GCTTTGACGTTGCCGACGCT
	sd-RD reverse	GTGGATTTCGGTTTGGTTCGCAT
	CG8509 forward	TTTTGCCGTATCTATGGGGTGC
	CG8509 reverse	TGCAGAGGCATGATCGGTACAA
	sd-RE forward	CTGCTGCTCTTGCTGTATTTTGC
	sd-RE reverse	GAAGCGAGAGAAACTCATCGAC
	sd-RE CDS forward	GTATGACTGGGTAATTCGGATGG
	sd-RE CDS reverse	GTCGTATTCCTATTCCTCCG
**Figure 3B**	Fab7 forward	AGGAAGAGAGCGGAAAGTGCA
	Fab7 reverse	CGGTCGCTCTTAGCCAATACTCTT
	Rp49 forward	ACCAGCTTCAAGATGACCATCC
	Rp49 reverse	CTTGTTCGATCCGTAACCGATG
**Figure 4A-C**	sd-RD forward	GCTTTGACGTTGCCGACGCT
	sd-RD reverse	GTGGATTTCGGTTTGGTTCGCAT
	CG8509 forward	TTTTGCCGTATCTATGGGGTGC
	CG8509 reverse	TGCAGAGGCATGATCGGTACAA
	sd-RE forward	GTATGACTGGGTAATTCGGATGG
	sd-RE reverse	GTCGTATTCCTATTCCTCCG
**Figure 4D**	PGRP Prom forward	TGAGCTTTCCAACACTCTTGC
	PGRP Prom reverse	GGTTTTGGTGGTTTATCTGAG
	sd-RD Prom forward	TTCTTCGATCCCTGCAAGGTCC
	sd-RD Prom reverse	GGAGAGCTGTGCGGATTGATTG
	CG8509 Prom forward	TGTCCAATTGCTCCGGGCATC
	CG8509 Prom reverse	ATATGCCTGCGAGCGAACCCA
	sd-RE Prom forward	CTGCTGCTCTTGCTGTATTTTGC
	sd-RE Prom reverse	GAAGCGAGAGAAACTCATCGAC
	sd 3'end forward	CGTAACCAGCCAGTAAGTATTG
	sd 3'end reverse	CCTTCTCCACCACTTGCTTG
**Figure S3A**	Fab7 forward	AGGAAGAGAGCGGAAAGTGCA
	Fab7 reverse	CGGTCGCTCTTAGCCAATACTCTT
	Rp49 forward	ACCAGCTTCAAGATGACCATCC
	Rp49 reverse	CTTGTTCGATCCGTAACCGATG
	Mini white forward	CTGCTTACCCACCCAAAACCAATCAC
	Mini white reverse	CGCTGCGTCCGCTATCTCTTTCGC
**Figure S3B**	CG32581 forward	GGAGCTTCCCAGCACATCAACC
	CG32581 reverse	TTGGAATCGAGGTACCCAGCG
	PGRP forward	TGGGTTACAACAGGATCTCGCT

Figure S5

PGRP reverse	ATATGTGACCATCCTCAACGCC
PRE 5'end forward	GGTAGTTCTGTTTATGCCATCG
PRE 5'end reverse	CCTCCACTACGGGTAAATTCC
Sd 1 forward	TTCTTCGATCCCTGCAAGGTCC
Sd 1 revese	GGAGAGCTGTGCGGATTGATTG
Sd 2 forward	GCTTTGACGTTGCCGACGCT
Sd 2 revese	GTGGATTTCGGTTTGGTTCGCAT
CG8509 forward	TTTTGCCGTATCTATGGGGTGC
CG8509 reverse	TGCAGAGGCATGATCGGTACAA
Sd 3 forward	CTGGGTGTGTGTGCGTGTGTAG
Sd 3 revese	GCAAAATACAGCAAGAGCAGCAG
Sd 4 forward	CTGCTGCTCTTGCTGTATTTTGC
Sd 4 revese	GAAGCGAGAGAAACTCATCGAC
CHC forward	GTGTGCAGATTGCCACCAAG
CHC revese	CGACCAGTTCGTCGGTTGAG
Graf forward	CGTACCCATGAGGGGATTTACAT
Graf reverse	AAGCAACAAATGAAGAGCGTAAC
CG32581 forward	GGAGCTTCCCAGCACATCAACC
CG32581 reverse	TTGGAATCGAGGTACCCAGCG
CG15602 forward	GTCTGGCCAATCTCACTGGGATG
CG15602 reverse	ATCGGAATCGACGGGTGTATC
PGRP forward	TGGGTTACAACAGGATCTCGCT
PGRP reverse	ATATGTGACCATCCTCAACGCC
sd-RD forward	GCTTTGACGTTGCCGACGCT
sd-RD reverse	GTGGATTTCGGTTTGGTTCGCAT
CG8509 forward	TTTTGCCGTATCTATGGGGTGC
CG8509 reverse	TGCAGAGGCATGATCGGTACAA
sd-RE forward	GTATGACTGGGTAATTCGGATGG
sd-RE reverse	GTCGTATTCCTATTCCTCCG
CHC forward	CGATCAAGTGAACAACCTGGAG
CHC reverse	CCGAAATGAACTCCTCCAGATC
shiX forward	CTCGGATTTGTGGAATCATCAG
shiX reverse	GTACTTTGACCTCTGACCTGACC
