# Supplementary material for: Polycomb Domain Formation Depends on Short and Long Distance Regulatory Cues
Source: PLoS One. 2013 Feb 20;8(2):e56531. doi: 10.1371/journal.pone.0056531 (PMC3577894; doi:10.1371/journal.pone.0056531)
Supplement: Figure S2 — Distribution of chromatin insulator binding sites at the sd gene locus. Annotated genes at the sd gene locus (X chromosome position 15628000 to 15668000) are shown on the top. Orange asterisk marks the transgene insertion site. Below, binding sites for the indicated insulator protein are shown [21]. (PDF) [file pone.0056531.s002.pdf]

Binding of known insulator proteins at the scalloped gene locusin wild type embryos

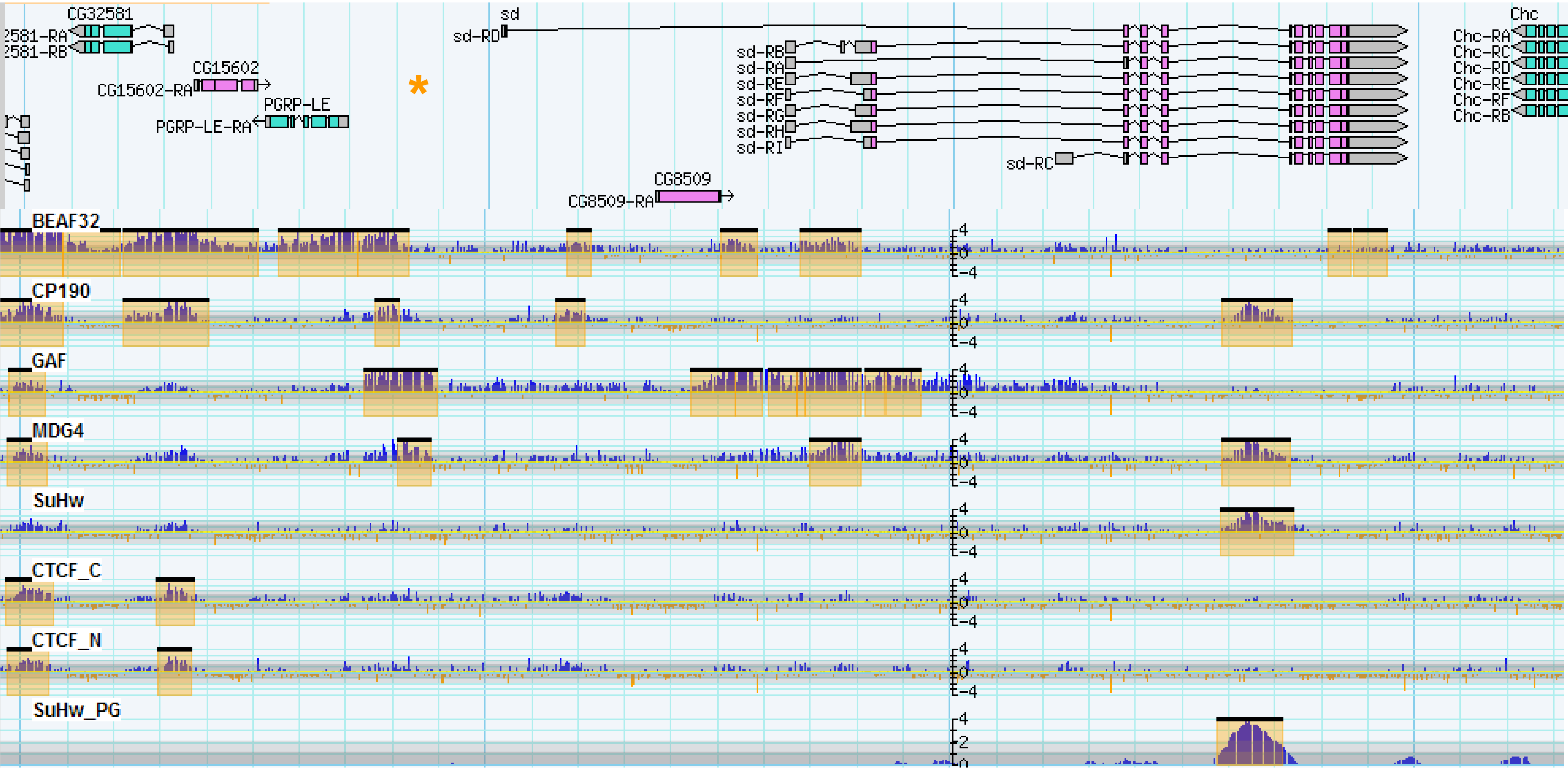

Figure S2
